# Life history and chemical ecology of the Warrior wasp *Synoeca septentrionalis* (Hymenoptera: Vespidae, Epiponini)

**DOI:** 10.1371/journal.pone.0194689

**Published:** 2018-03-22

**Authors:** Eliaber B. Santos, Sue Shemilt, Carlos A. L. de Carvalho, Stephen J. Martin

**Affiliations:** 1 Universidade Federal do Recôncavo da Bahia, Rua Ruí Barbosa, 710—Centro, Cruz das Almas—BA, Brazil; 2 Chemical Ecology Group, School of Physical and Geographical Sciences, Lennard-Jones Laboratory, Keele University, Keele, United Kingdom; 3 School of Environment and Life Sciences, The University of Salford, Manchester, United Kingdom; Universidade de Sao Paulo Faculdade de Filosofia Ciencias e Letras de Ribeirao Preto, BRAZIL

## Abstract

Swarm-founding ‘Warrior wasps’ (*Synoeca* spp.) are found throughout the tropical regions of South America, are much feared due to their aggressive nest defence and painful sting. There are only five species of *Synoeca*, all construct distinctive nests that consist of a single sessile comb built onto the surface of a tree or rock face, which is covered by a ribbed envelope. Although locally common, research into this group is just starting. We studied eight colonies of *Synoeca septentrionalis*, a species recently been described from Brazil. A new colony is established by a swarm of 52 to 140 adults that constructs a colony containing around 200 brood cells. The largest colony collected containing 865 adults and over 1400 cells. The number of queen’s present among the eight colonies varied between 3 and 58 and no clear association between colony development and queen number was detected. Workers and queens were morphologically indistinguishably, but differences in their cuticular hydrocarbons were detected, particularly in their (Z)-9-alkenes. The simple cuticular profile, multiple queens, large size and small number of species makes the ‘Warrior wasps’ an excellent model group for further chemical ecology studies of swarm-founding wasps.

## Introduction

In tropical regions, the independent founding Vespine wasps (hornets [*Vespa*] and yellow-jackets [*Vespula*]) are replaced by the swarm-founding Polistinae. In the Neo-tropics, these swarm-founding wasps have undergone a spectacular radiation. So, although only 25% of Polistinae species are swarm-founding, they are the most taxonomical diverse, exhibit the greatest diversity of nest structures and range of colony sizes [1, 2]. This makes swarm-founding wasps one of the most highly successful and diverse groups of social insects, but despite this, they remain a poorly studied group.

Swarm-founding wasps are of particular interest as their colonies contain multiple queens, rather than the single queen normally found in temperate Vespine wasps. Furthermore, there is often no obvious caste dimorphism between the queens and workers in swarm-founding species, since both castes (queens and workers) can be morphologically identical or very similar. Maintaining an effective colony structure were nest-mates can be derived from tens or even hundreds of queens may be potentially difficult, due to an increase in genetic diversity. One solution is "cyclical oligogyny", where in the swarming founding wasp *Metapolybia aztecoides* the queen number varies over time and prior to sexual production the number of queens in the colony falls to a low number [3]. This change is behaviourally mediated and similar interactions between nest-mates (queens and workers) have been shown in the Epiponini wasps *Asteloeca ujhelyii* [4] and *Synoeca surinama* [5] another caste-flexible swarm-founding wasp closely related to the study species. However, in other species such as the thelytokous ant *Cerapachys biroi*, nest-mates synchronously alternate between reproduction and brood care [6]. Whatever, the actual mechanism is, it requires a detailed chemical recognition system to exist, so the various nest-mates (queens, workers and males) can be recognised and the appropriate behaviours performed. It is now well established that nest-mate, caste and task recognition is mediated via cuticular hydrocarbons (CHC) found on the surface of all insects [7].

The ‘Warrior wasps’ belong to the *Synoeca* genus (Polistinae) and are a large (individual wasp size) swarm-founding social wasps [8] that has the potential to be a good model system for chemical ecology studies. The genus consists of just five species (*Synoeca chalibea; S*. *virginea; S*. *surinama; S*. *septentrionalis* and *S*. *cyanea)* [9], are believed to have evolved in the Amazon [10] and are now found throughout Central and South America. All species have a distinctive blackish or dark blue metallic-like appearance and all built a distinctive nest structure [1]. The nest consists of a single sessile brood comb built directly onto the surface of a tree or rock [11], which is covered by a single layer of envelope. The envelope has a distinct ribbed surface and a single entrance hole that top of the nest (see results). Most nests have between one and three lobes, but nests with nine lobes spanning over three meters have been reported. Warrior wasps are much feared by the local people due to their defensive behaviour that involves drumming on the nest surface [12], followed by a mass attack it the threat persists. The sting is extremely painful and is given the top level of ‘4’ on the ‘Schimdt pain scale’ [13]. Nests are relatively easy to find and fairly common throughout its range. The only chemical ecology study on this group [5] indicated a very simple cuticular hydrocarbon (CHC) profile consisting of only *n*-alkanes and alkenes, a profile type found in less than 20% of social wasps [14]. This type of simple CHC profile led to the discovery of the nest-mate recognition system in the Narrow-headed ant *Formica exsecta* [15].

The aim of this study was to investigate the basic life-history and chemical ecology of the ‘Warrior wasp’ *S*. *septentrionalis* colonies in North East Brazil, a species only recently recorded in Brazil [11]. The central question was to look for any morphological or chemical differences between the queens, workers or males.

## Materials and methods

### Colony collection

**Fig 1:**
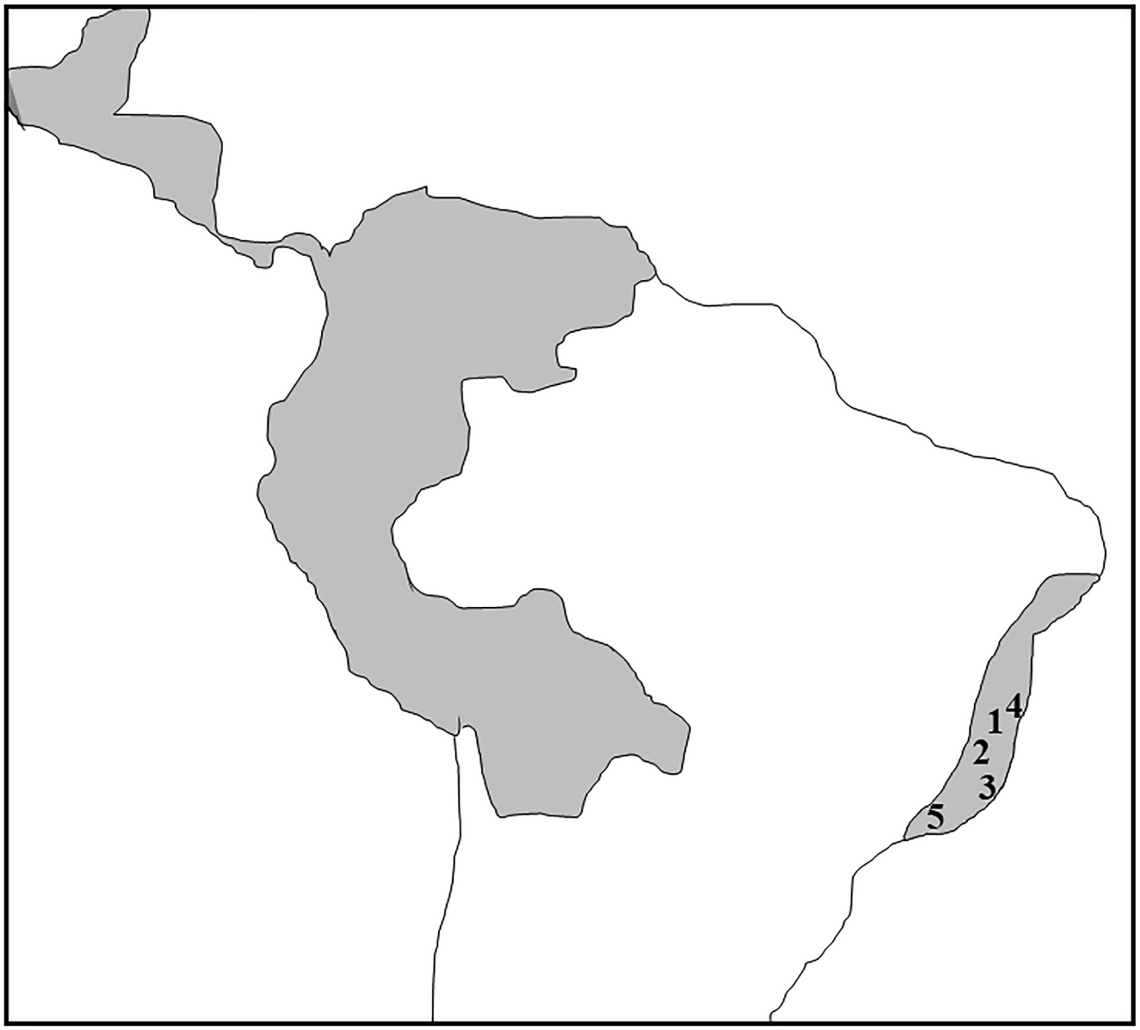
The known distribution of *Synoeca septentrionalis* in South America. This is shown in grey with the two study sites in state of Bahia, 1 = Cruz das Almas and 2 = Mutuipe, and previous published records [11] from Bahia at 3 = Itabuna and 4 = Santa Terezinha, also 5 = Alfredo Chaves in the state of Espírito Santo.

Nine active colonies of *S*. *septentrionalis* where studied, three (MU-1, 2 & 3) from Muiepi (altitude 300m) and six (UN-1 to UN-6) from Cruz des Lams (altitude 200m) both in the state of Bahia, NE Brazil (Fig 1).

**Fig 2:**
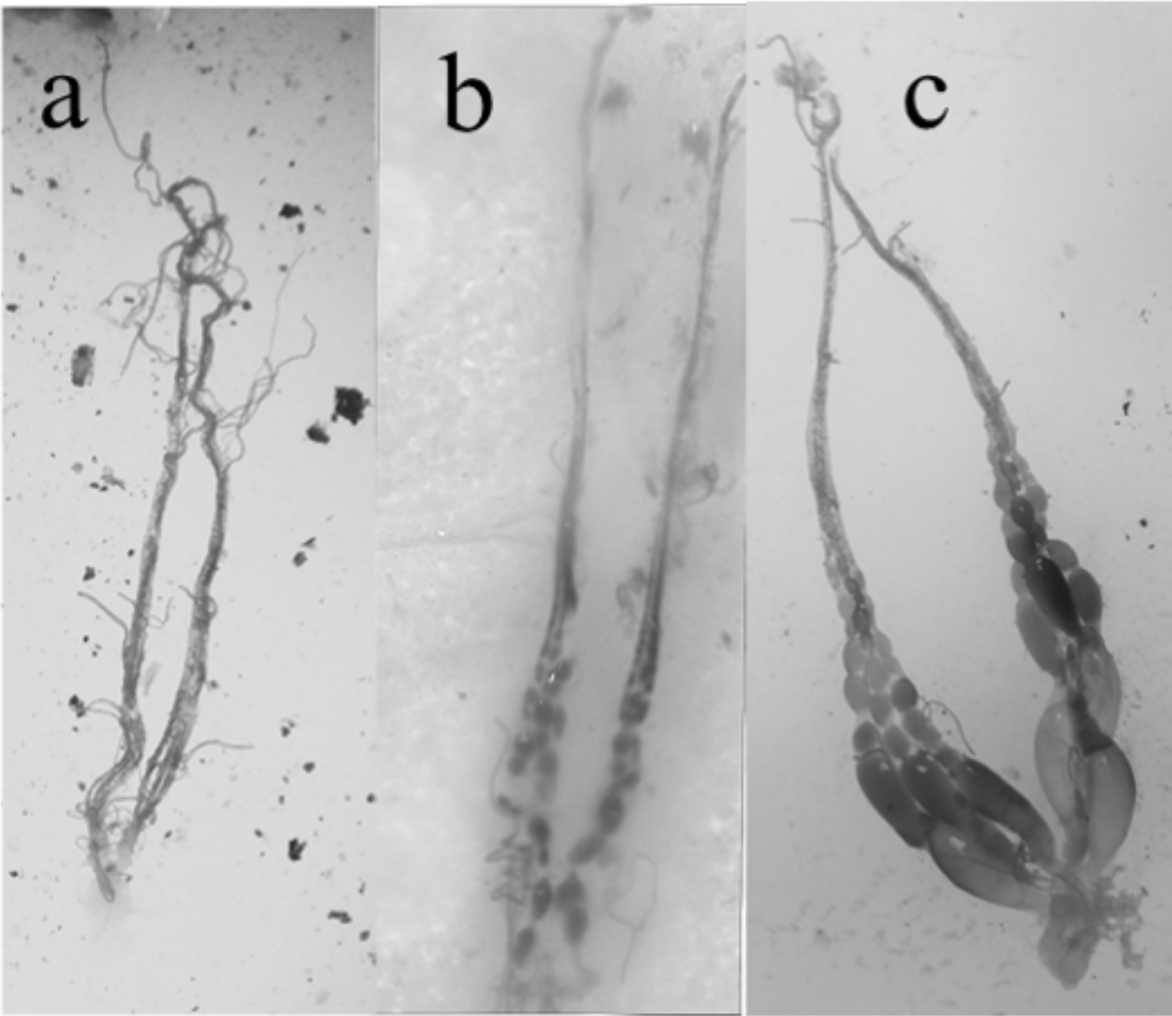
Images of typical different stages of ovary development in *Synoeca septentrionalis*. This was used to classify the females as a) workers = filamentous ovaries, b) intermediates = small eggs or c) queens = mature eggs.

All nests were collected between 7-Jan-2015 and 12-Sept-2016, at night by sealing the entrance with tissue paper and injecting chloroform into the nest. After collecting the adults, a large knife was used to remove the single sessile comb from the surface of the tree. The adults were then frozen and species identification confirmed using published keys [9, 11]. The age of the colonies was unknown, since the date when the colony was established was not known in most cases. Each colony was classified as pre-emergent or post-emergent, based on if the first batch of adults had emerged from the brood comb or not. For each colony the number of eggs, larvae, pupae and empty cells (raised, or newly constructed) were recorded on a ‘cell map’ that was later visualised using CoralDRAW X8. The number of adult males and females were classified using the presence of genitalia or a sting. Then all females were dissected to determine the developmental state of their ovaries (Fig 2) and classified into, workers (filamentous ovaries), intermediates (presence of small eggs) or queens (mature eggs and enlarged ovaries). We did not check if females were inseminated or not.

### Morphometric analysis

**Fig 3:**
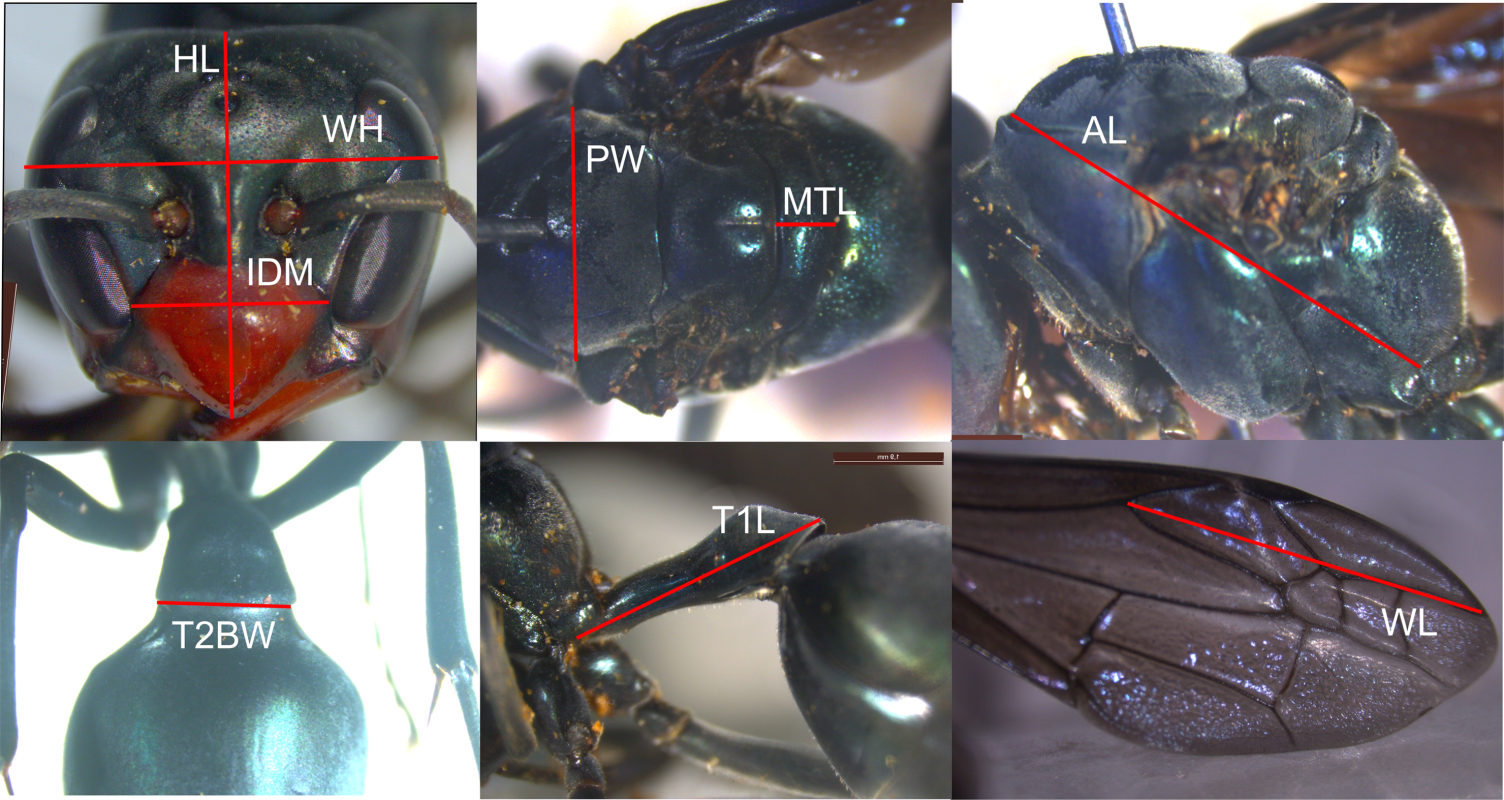
The nine morphometric measurements recorded. HL = head length, HW = head width, IDM = gena width, PW = pronotal width, MTL = mesoscutellar length, AL = alitrunk length, T2BW = basal width of tergite II, T1L = length of tergite I, and WL = partial length of forewing.

Adults wasps (queens, workers and males) from three colonies (MU-1, UN-1, & UN-2) had nine external body parts (Fig 3) measured using a Lecia binocular microscope (x12.5) fitted with a Lecia camera. The measurements used were based on a morphometric study of several swarm-founding species [4]. Only the measurements of adults from the first colony were done prior to the dissections i.e. blind. However, due to the large number of adults in the other two colonies they were firstly classified into their groups via dissection, then all queens, males and a randomly chosen sub-group of workers were measured. All comparisons between the three groups (queens, workers and males) were conducted using a one-way ANOVA followed by a post hoc test when required.

### Chemical analysis

The same three colonies subject to morphometric analysis (MU-1, UN-1 and UN-2) also had their cuticular hydrocarbon profiles (CHC) analysed. From each wasp one pair of wings were removed using fine scissors prior to dissection and placed individual into a glass vial into which a 100μl of high-performance liquid chromatography (HPLC) grade hexane was added, so immersing the wing in the liquid. After ten minutes, the wings were removed and sample evaporated to dryness at room temperature in a fume cupboard, before being stored at -20°C. The wings are used as they provide an accurate representation of the CHC profile of the individual without any potential contamination from various glands that open onto the surface of the body [16, 17].

Immediately before analysis, samples were re-suspended in 30μl HPLC grade hexane and analysed on an Agilent 7890-GC (equipped with an Vf-5ht UltiMetal column; length: 30m; ID: 0.25mm; film thickness: 0.1μm) connected to an Agilent 5975-MSD (quadrupole mass spectrometer with 70-eV electron impact ionization). Samples were injected in splitless mode, with the injection port at 325°C and the MS in scan mode. The oven temperature programme was 70°C (held for 1 minute), 40°C min^-1^ to 200°C, 4°C min^-1^ to 250°C and finally 25°Cmin-1 to 350°C with a final five-minute hold. The carrier gas helium was used at a constant flow rate of 1.0ml min-1. Compounds were identified using standard MS databases, diagnostic ions and Kovats indices. This method allowed us to detected hydrocarbons up to carbon chain lengths of forty-two carbons (C42). The peaks were integrated manually using Agilent MSD ChemStation before converting the values into percentages, either using all the compounds, or just using the *n*-alkanes or alkenes. The data was then visualized using histograms in order to search for patterns. The alkene double bond positions were determined using dimethyl disulfide (DMDS) derivatization [18]. From two colonies, pooled wing extracts from five workers and five queens were subjected to DMDS derivatization. The four samples were re-analysed on the GC-MS under similar conditions as the non-derivatizated samples.

## Results

**Fig 4:**
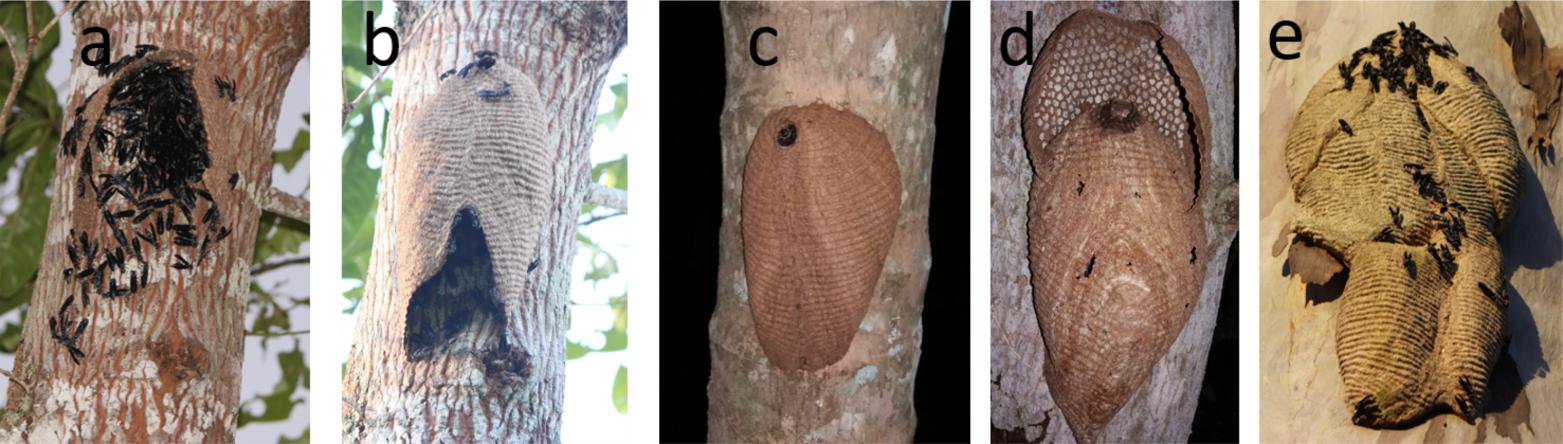
Progression of colony development in the Warrior wasp *Synoeca septentrionalis*. From the a) the initial nest establishment, b) closing the envelope, c) a young pre-emergent single lobed colony to d) nest expansion via the building of an additional lobe, to a e) large mature six-lobed nest.

All nine colonies were located at one to five meters above the ground on a variety of smooth surfaced trees, although colonies up to heights of 10m were seen. One swarm (UN-6) of around 140 adults were seen on the surface of a tree on 2^nd^ Oct-2016, three days later the sessile comb pattern was established and envelope partly built (Fig 4A), then on 9^th^ Oct the envelope was almost completed (Fig 4B) but, the nest was destroyed before it could be collected. The nine study colonies represent four in the pre-emergent phase and five in the post-emergent phase (Table 1).

**Table 1:** The colony details of the nine study colonies of *Synoeca septentrionalis* collected from NE Brazil. The colonies are ordered in those that are pre-emergent (Pr) and post-emergent (Po).

Date collected	Colony	Lat	Long	Cell total	Eggs	larva	pups	empty cells	Adults	Queen	Inter	Male	Worker
08/09/2016	UN6 Pr	12.660262	39.084561						~140				
11/04/2016	UN3 Pr	12.656931	39.087120	175	113	0	0	62	130	3	85	0	41
12/09/2016	MU3 Pr	13.349587	39.536151	117	22	73	0	22	80	23	19	0	38
11/09/2016	MU2 Pr	13.347218	39.541863	182	18	102	28	34	52	10	0	3	39
11/04/2016	UN4 Po	12.656842	39.086830	254	67	102	85	10*	141	25	16	0	100
19/01/2015	UN1 Po	12.651472	39.052528	518	143	144	192	39	168	33	12	20	103
07/01/2015	MU1 Po	13.345535	39.542585	532	132	217	138	45	89	6	0	8	75
26/07/2016	UN5 Po	12.657079	39.087294	1020	242	156	231	391	359	45	11	162	177
09/07/2015	UN2 Po	12.660262	39.084561	1416	300	304	409	403	865	58	76	2	729

* in colony UN-4, 185 newly built empty cells form the new nest extension (see Fig 4D).

### Pre-emergent stage

A new colony of *S*. *septentrionalis* is established by a swarm containing between 52 to 140 adults. The swarm firstly constructs the base of the comb, consisting of around 200 cells, then around this the envelope is built (Fig 4C). The envelope extends well below the comb so allowing space for some further comb expansion, without removing and replacing the envelope (Fig 4D). However, when colony expansion occurs it is achieved by building a new comb above the existing colony and covering this with a new lobe of envelope. So, the colony grows upwards, although in colony UN-2 the width of the tree allowed new lodes to be added above and to the sides of the original colony (Fig 4E), with up to six lobes being visible. It was estimated that no pre-emergent colony was more than one month old.

### Post-emergent stage

**Fig 5:**
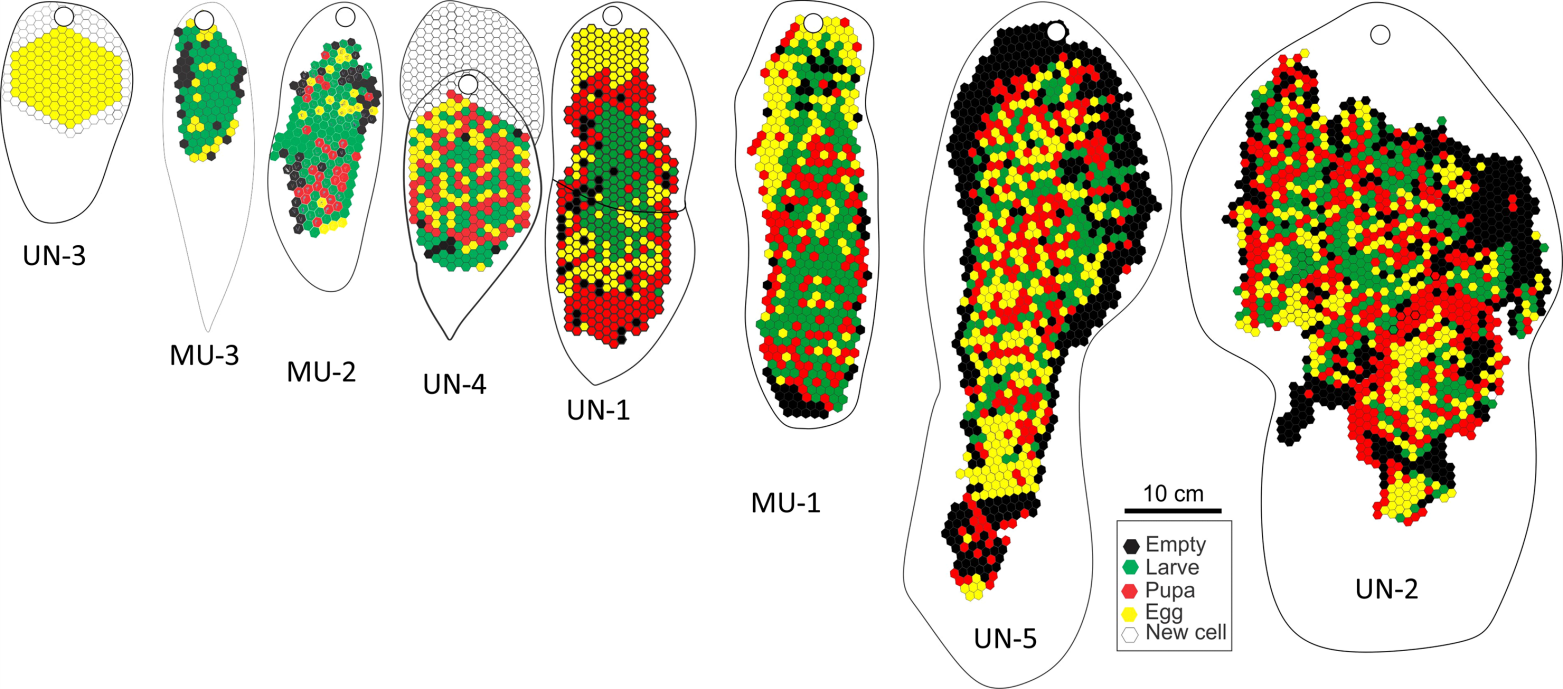
Cell maps and envelop outlines of the eight *Synoeca septentrionalis* study colonies arranged in order of size and probably age. The colony codes correspond to those in Table 1. White = empty shallow cell, Black = empty deep cell, yellow = eggs, green = larva and red = pupa. All colonies are to scale.

The post-emergence colonies are larger, consist of multiple lobes, contain more adults (89–865) and many more brood cells (Table 1). The number of queens varies between 3 and 58 and shows no clear pattern, as do the number of intermediates or males within the colonies. The decrease in worker numbers during the pre-emergent stage i.e. after swarming, and before the emergence of the new workers may reflect the loss of older workers. The brood pattern is uniform in the younger nests but as cells are reused the clear brood patterns break down (Fig 5).

### Morphological caste differences

No consistent significant differences (all values p >0.05) were found between the 41 queens and 124 workers in *S*. *septentrionalis* in any of the three colonies studied (S1 Table). However, the males had significantly smaller head measurements and shorter wing measurements (all values p <0.05) (S1 Table). All individuals (queens, workers and males) from colony MU-1 were noticeable smaller than those from colonies (UN-1and UN-2) (S1 Table), but males again were clearly different in size from the workers and queens (S1 Fig). The species identification of all colonies was re-confirmed.

### Chemical analysis

**Fig 6:**
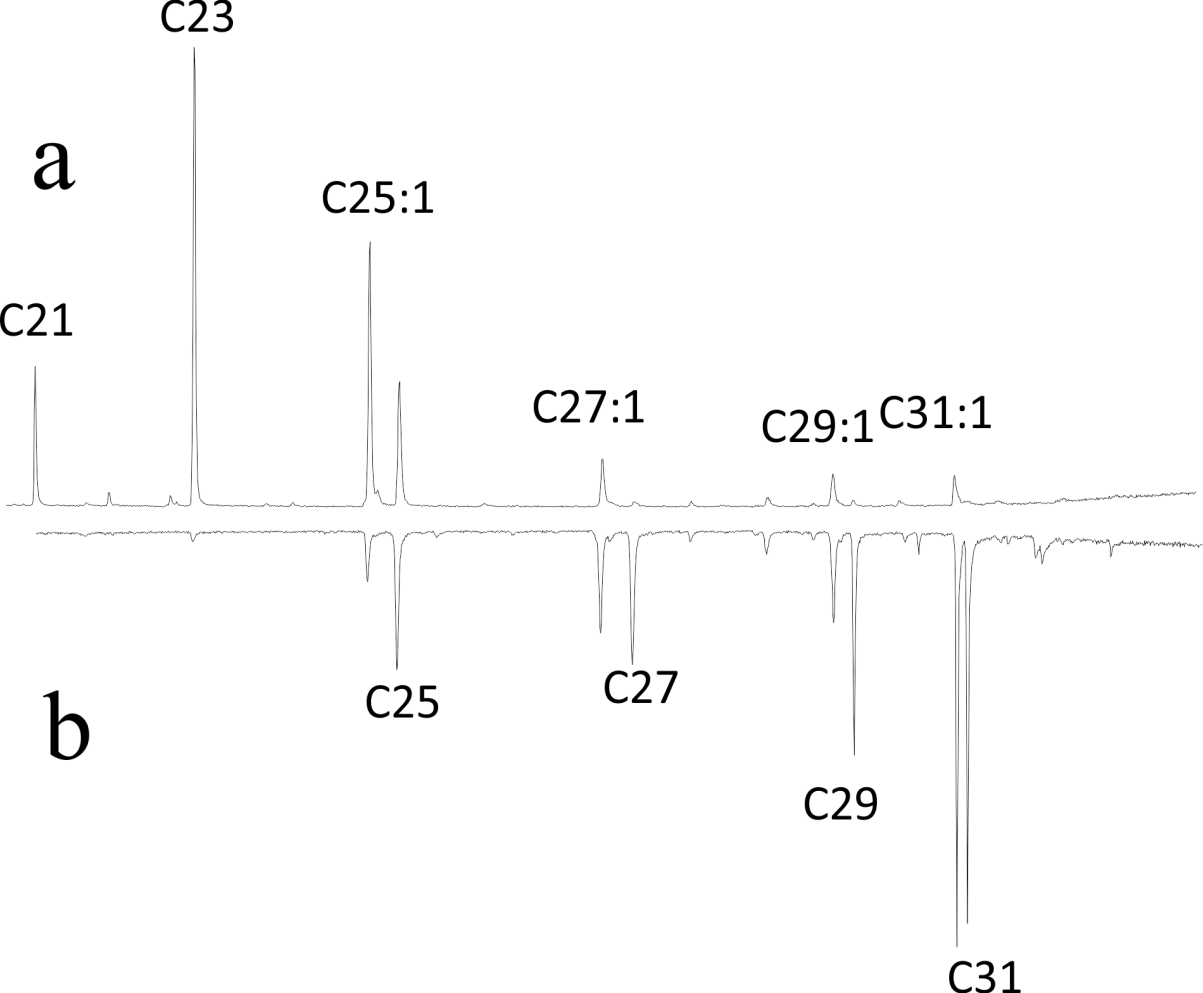
Typical total ion chromatograms of *Synoeca septentrionalis*. Showing a) newly emerged callow and b) the inverted worker profile indicating the shift from the short-chained to longer-chained profiles as the wasps ages. All alkenes (Cn:1) are the (Z-9) isomer.

We obtained 130 high quality Total Ion Chromagrams from *S*. *septentrionalis* adults (queens, workers and males), all from post-emergent colonies (MU-1, UN-1, & UN-2). The CHC profile of all adults consisted of a series of *n*-alkanes (C21 to C33) and (Z)-9-alkenes (C25:1 to C33:1) (Table 2). An initail investigation of the data indicated that both colony and caste differences in the CHC profiles were present, so the data from each colony and caste was treated separately. Furthermore, two distinct types of CHC patterns were seen in all colonies. An individual was either dominated by shorter chained CHC’s e.g. C21, C23 & (Z)-9-C25:1, or these were replaced by a predominance of longer CHC’s C27, 29 & 33 n-alkanes and (Z)-9-alkenes. This pattern was also seen in *S*. *surinama* [10] and supported our idea that is profile is associated with age, since the profiles of newly emerged workers (emerged after nest collection) were very distinct (Fig 6) dominated (>80%) by short chained CHC (C21, C23 & [Z]-9-C25:1), whereas all queens and the vast majority of workers or males these compounds contributed less than 20% of the profile, just as seen in *S*. *surinama* [5]. Therefore, eight males and eleven workers from a total of 130 adults were analysed separately as putative recently emerged adults since 30–80% of their CHC profile was made up of these three CHC.

**Table 2:** The proportions of CHC’s extracted from the wings of different castes and sexs repeated across three colonies. The mean precentgaes and SD of each compound excrated from queens, workers and males from each study colony. Also the overall precentage of *n*-alkanes and alkenes, present in each group is given. The precentage is calculated using the total amount of CHC ions present in the sample.

	UN-1	UN-1	UN-1	MU-1	MU-1	MU-1	UN-2	UN-2
	Q = 22	W = 22	M = 19	Q = 6	W = 12	M = 6	Q = 19	W = 10
C23	0.6±2.4	1.0±0.3	4.4±1.0	4.0±1.4	9.9±8.0	8.1±7.8	0.5±0.6	0.9±0.5
(Z)-9-C25:1	8.1±3.5	3.8±2.9	7.9±1.6	16.8±6.1	15.6±7.4	13.4±8.1	4.9±1.7	3.5±2.1
C25	25.9±1.9	11.0±4.0	18.2±6.6	29.3±6.6	20.1±6.0	18.7±3.6	23.6±2.7	13.2±3.6
(Z)-9-C27:1	2.1±1.7	9.8±0.7	7.5±11.3	6.3±2.4	10.6±2.8	12.9±2.7	2.8±1.7	9.5±4.2
C27	9.0±3.5	13.6±1.0	11.2±4.3	10.4±1.4	6.9±4.0	8.4±4.1	7.8±1.1	13.5±3.2
(Z)-9-C29:1	2.3±0.7	6.3±0.7	7.8±1.5	6.1±3.1	9.2±2.3	10.9±2.0	3.9±3.2	10.7±2.5
C29	16.9±2.1	13.7±3.0	11.6±3.3	10.0±2.1	7.0±3.8	7.2±3.7	15.3±1.9	10.3±2.8
(Z)-9-C31:1	8.1±1.8	15.5±2.6	14.9±3.9	9.3±3.1	13.5±5.0	13.3±3.4	13.5±3.2	20.8±5.3
C31	26.6±1.8	21.2±5.6	14.1±4.8	7.2±1.9	5.6±3.6	5.3±3.3	26.7±5.5	14.5±4.9
C33:1	0.1±0.6	1.7±0.2	1.1±0.6	0.4±0.5	1.0±0.6	1.1±0.4	0.5±0.5	1.8±0.8
C33	0.3±0.6	2.3±0.3	1.3±0.7	0.3±0.3	0.6±0.4	0.5±0.3	0.6±0.4	1.5±0.8
% Total Alkanes	79.3	62.8	60.8	61.1	50.1	48.3	74.5	53.8
% Total Alkenes	20.7	37.2	39.2	38.9	49.9	51.7	25.5	46.2

**Fig 7:**
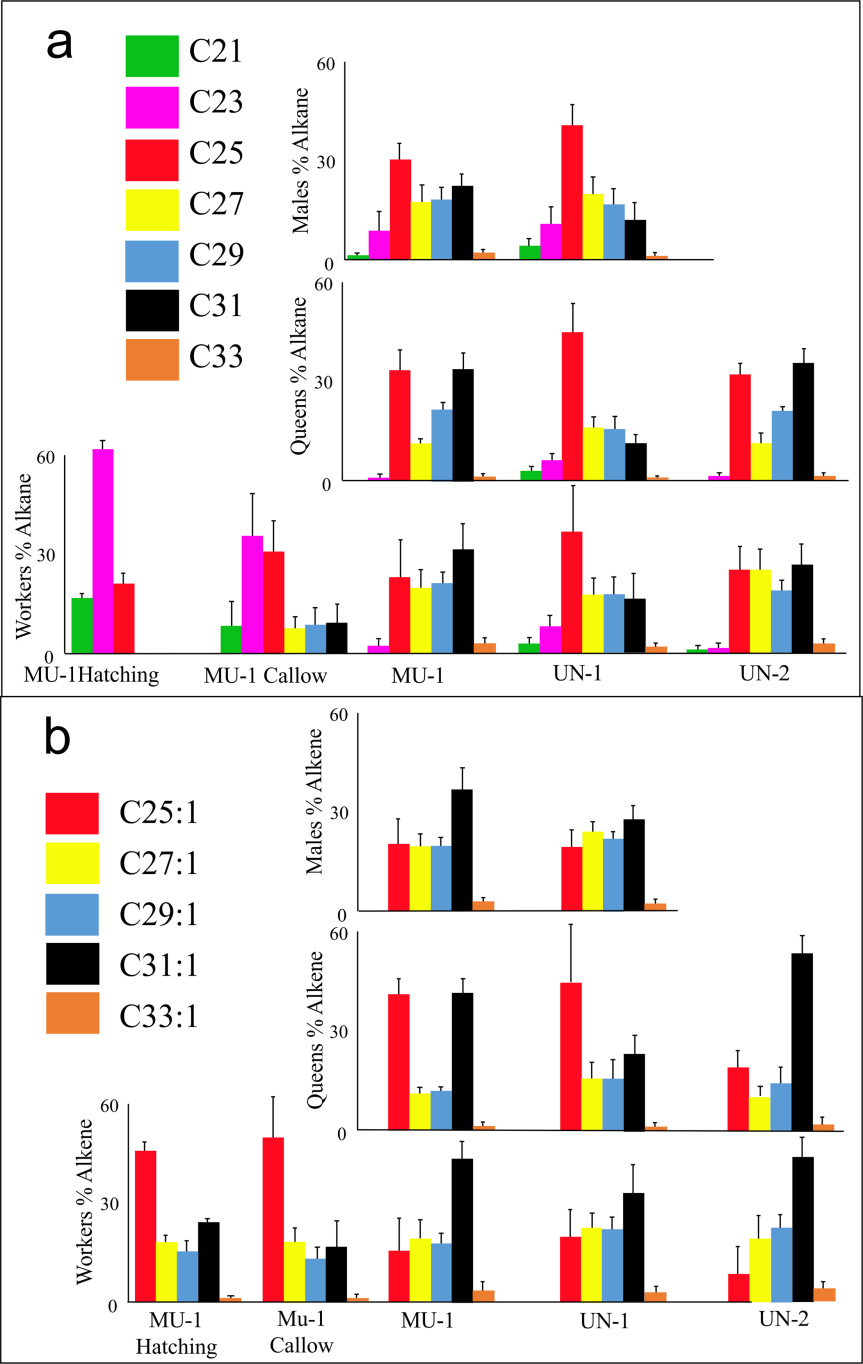
The percentage of *n*-alkanes and alkenes in each colony and group studied when considered separately. The profiles of a) *n*-alkanes and b) (Z)-9-alkenes of the just emegered callow, worker, queen and males in each of the three study colonies (MU-1, UN-1 & UN-2) when precentages are calculated based on only the total amount of ions in that group. The error bars represent one SD.

We found no qualtative differences between the castes or colonies, although quntitative differences were present. However, there was no clear pattern that was unique to either a colony or a caste (Table 2). In each colony the relative proportion (%) of *n*-alkanes in queens was always greater than in the workers or males (Table 2) within a colony. We then separated out the *n*-alkanes and alkenes and reanalysing them again. Since there are many studies that indicate that alkenes consistantaly elicate a much stronger behavioural response in aggesion bioassays than linear-alkanes [15, 19, 20] suggesting that communication is via the alkene profile, while the alkane’s function as anti-dessication molecules. This revealed that all workers and males shared a similar CHC profile (Fig 7). That is the alkane and to a lesser degree the profile for workers and males within a colony are similar, whereas, between colonies they can be more varabile. The queens had a distinctive profile with peaks at (Z)-9-C25:1 and (Z)-9-C31:1, although some colony varaiation did exist. The most interesting observation was the dramatic change in the *n*-alkane profile from the callows to the older adults with the shorter chained alkanes (C21 & C23) been almost entirely replaced by longer chained alkanes. However, the changes in the alkene profile from callow or newly emerged adults were not as dramatic. Furthermore, the ratios of the total ion counts of (Z)-9-C27:1, (Z)-9-C29:1 and (Z)-9-C31:1 were all highly correated (r^2^>0.8) so suggesting they production is tightly regulated, whereas, (Z)-9-C25:1 production is not correlated to any other alkene, indicating the production of (Z)-9-C25:1 and the other alkenes are independent, the reason for this remains unknown.

## Discussion

Until recently *S*. *septentrionalis* was not recorded from Brazil being found in Central America (Mexico to Panama) and the NW countries of South America (Colombia, Venezuela, Ecuador, Peru and Bolivia) [1, 9]. However, recently the collection of two *S*. *septentrionalis* nests from the Atlantic rain forest in the states of Bahia and Espírito Santo (Fig 1) [11] considerably extended the range of this species. This study found *S*. *septentrionalis* to be the most common *Synoeca* species in the study area of Bahia, with only one *S*. *suriname* colony found against the nine *S*. *septentrionalis* colonies located during this study. This suggests that many previous records from the NE region of Brazil may have been misclassified *S*. *septentrionalis* as *S*. *suriname* or *S*. *cyanea*, since these are morphological similar species and the reddishness of the clypeus is a variable character [10]. So, the additional external morphological features such as the deep punctuation on the head; erect setae on the scape and erect setae on the scutum mentioned by [10] are vital for correct species identification.

Although, this is the first study on *S*. *septentrionalis* colonies, comparisons with previous studies on *S*. *surinama* [5, 21–24] and *S*. *cyanea* [8] colonies, indicate that these two-other species are similar in size and queen number is highly variable in all three species (Table 3). No clear pattern between, the number of males, queens and colony cycle was detected in any of the studies. Therefore, it is not possible to determine if any species of *Synoeca* undergoes cyclical oligogyny, as suggested for other Epiponini wasps [3, 4], although very low queen numbers were present in some nests, which suggests this may be happening. During the pre-emergent stage and in smaller colonies clear brood patterns can be seen, but these are lost in larger colonies as cells are reused and brood cannibalism prior to swarming has been observed [23]. New nests can be established at any time of the year, which will be helped by the lack any distinct seasons in the study area. As the number of adults in the swarm (52–140) are normally much less than the number in the post-emergent colonies, reproduction by only colony budding can be assumed. Although the very large numbers of adults in pre-emerged nests in *S*. *cyanea*, suggest that an entire colony re-located, possibly due to their previous colony been destroyed or disturbed.

**Table 3:** Comparison of basic colony characteristics for three *Synoeca* spp all collected from Brazil.

	No. of adults Pre-emergent	No. of adults Post-emergent	No. of queens Pre-emergent	No. of queens Post-emergent	No. of cells	Ref.
*S*. *septentrionalis*	52–139	89–865	3–23	6–58	117–1416	This study
*S*. *surinama*	40–350	approx. 1000				[23]
*S*. *surinama*					307–2151	[21]
*S*. *surinama*		12–861		0–59 (mean = 16)		[22]
*S*. *surinama*		1474		103		[24]
*S*. *surinama*	200	65–300	12	1–32		[5]
*S*. *cyanea*	58–830	1170–1279	1–16	42–84	1200	[8]

All nests of *Synoeca* were constructed at heights between 1m to over 6m. The nest construction is unusual among social wasps, since the base of the sessile comb is built first then the walls of the envelope are constructed. The ribbed structure results from the extension of the edge effect of hexagonal shaped comb cells [21]. This also explains why when the nest is extended by building a new comb base, outside the existing nest prior to the envelope removal, the comb between the two sections join perfectly.

This study found no significant morphological differences between queens and workers in *S*. *septentrionalis*, just like in *S*. *chalibea* [1], *S*. *surinama* [1, 24] and *S*. *cyanea* [8]. However, we did find a significant morphological difference between the females and males. The males had a significantly smaller head and shorter wings, although other areas i.e. thorax and tergite was the same size as females. A comparison of the measurements indicates that *S*. *septentrionalis* is a similar size than *S*. *cyanea* (correcting for the x10 error in the measurements in) [8] and both these species appear to be around 20% bigger than its sister species *S*. *surinama* using the measurements in [24]. However, a colony of *S*. *surinama* collected from the study area had workers that were clearly larger than *S*. *septentrionalis* suggesting a large variation in body size among each species, although within a colony, sizes are very consistent.

Very little is known about the chemical ecology of the *Synocea* group, with only one previous study [5] on *S*. *surinama*. Both species of *Synocea* had a CHC based on a series of C21-C33 *n*-alkanes and (Z)-9-alkenes, and dienes although the double bond positions have only been determined in *S*. *surinama* as the Z9 [5], i.e. the same as *S*. *septentrionalis* (this study). The presence of olefins (alkenes & dienes) in social wasps is unusual since wasps have predominantly diversified their methyl-branched alkanes production [14], such as found in the swarm founding neo-tropical wasp *Mischocyttarus cassununga* whose CHC is dominated by mono and di-methyl-alkanes [25], typical of most Vespidae [14].

Within, the *S*. *septentrionalis* CHC data both caste and colony differences occur, these are more distinct in the alkenes, but difficult to understand within a limited number of colonies sampled. Although, in both *S*. *septentrionalis* (Fig 7) and *S*. *surinama* [5] (Z)-9-C25:1 was consistently higher in the queens than workers in all colonies. In some, but not all, colonies (Z)-9-C31:1 was higher in queens than workers, and in general the workers tend to have a higher proportion of longer hydrocarbons (e.g., C32, C33:1, and C33) in both species. Although, the newly emerged workers ‘callows’ had a CHC profile dominated by C23, (Z)-9-C25:1, C25, and (Z)-9-C31;1, a pattern very similar to that of a queen. The subsequent changes seen in *S*. *septentrionalis* between the CHC in the callows (and putative newly emerged adults) and workers, has also been reported in other social wasp species, including *S*. *surinama* [5], *Polistes dominulus* [26], *P*. *fuscatus* [27] and *Polybia micans* [28] as well as in the honey bee *Apis mellifera* [29, 30], indicating this may be a widespread phenomenon among social insects.

## Supporting information

S1 Table Morphometric analysis (mean ± SD) of *Synoeca septentronalis* queens, workers and males from three colonies. (DOCX)

S1 Fig Discriminate analysis of *Synoeca septentronalis* adults. (DOCX)
