# Supplementary material for: Life history and chemical ecology of the Warrior wasp Synoeca septentrionalis (Hymenoptera: Vespidae, Epiponini)
Source: PLoS One. 2018 Mar 22;13(3):e0194689. doi: 10.1371/journal.pone.0194689 (PMC5864055; doi:10.1371/journal.pone.0194689)
Supplement: S1 Table — (DOCX) [file pone.0194689.s001.docx]

**S1 Table. Morphometric analysis (mean ± SD) of *Synoeca septentronalis* queens, workers and males from three colonies**. The measurements are given in mm and analysed using a One-Way ANOVA with Tukey post hoc test that showed that the only constant different between the three groups, was males had significantly smaller IDM and WL measurements.

|  | MU1 | MU1  Worker n=75 | MU1  Male n=8 | UN1 | UN1 | UN1 | UN2 | UN2 | UN2 |
| --- | --- | --- | --- | --- | --- | --- | --- | --- | --- |
|  | Queen n=6 |  |  | Queen n=23 | Worker n=19 | Male n=18 | Queen n=26 | Worker n=26 | Male n=2 |
| Head |  |  |  |  |  |  |  |  |  |
| HL | 4.34 ± 0.08 | 4.45 ± 0.11 | 3.99 ± 0.10 | 4.88 ± 0.11 | 4.89 ± 0.13 | 4.31 ± 0.15 | 4.71 ± 0.16 | 4.77 ± 0.11 | 3.96 ± 0.11 |
| HW | 4.98 ± 0.08 | 4.95 ± 0.11 | 4.57 ± 0.13 | 5.11 ± 0.07 | 5.12 ± 0.13 | 4.65 ± 0.13 | 4.99 ± 0.10 | 5.06 ± 0.19 | 4.54 ± 0.00 |
| IDM* | 2.38 ± 0.04 | 2.39 ± 0.07 | 1.79 ± 0.07 | 2.48 ± 0.06 | 2.51 ± 0.06 | 1.87 ± 0.06 | 2.42 ± 0.06 | 2.49 ± 0.07 | 1.81 ± 0.05 |
| Mesossoma | |  |  |  |  |  |  |  |  |
| PW | 4.10 ± 0.18 | 4.09 ± 0.16 | 4.05 ± 0.18 | 4.18 ± 0.10 | 4.25 ± 0.11 | 4.13 ± 0.20 | 4.14 ± 0.11 | 4.28 ± 0.13 | 4.09 ± 0.00 |
| MTL | 1.61 ± 0.05 | 1.59 ± 0.07 | 1.60 ± 0.07 | 1.67 ± 0.07 | 1.71 ± 0.05 | 1.58 ± 0.16 | 1.63 ± 0.06 | 1.55 ± 0.08 | 1.57 ± 0.01 |
| AL | 7.18 ± 0.27 | 7.03 ± 0.39 | 6.64 ± 0.23 | 7.10 ± 0.15 | 7.01 ± 0.24 | 6.91 ± 0.33 | 6.83 ± 0.20 | 6.85 ± 0.27 | 6.75 ± 0.12 |
| Metassoma | |  |  |  |  |  |  |  |  |
| T2BW | 2.18 ± 0.08 | 2.23 ± 0.10 | 2.17 ± 0.12 | 2.34 ± 0.10 | 2.40 ± 0.15 | 2.33 ± 0.17 | 2.30 ± 0.07 | 2.41 ± 0.50 | 2.28 ± 0.09 |
| T1L | 4.76 ± 0.08 | 4.81 ± 0.25 | 4.55 ± 0.34 | 5.00 ± 0.24 | 5.10 ± 0.19 | 4.92 ± 0.22 | 4.82 ± 0.16 | 4.88 ± 0.19 | 4.75 ± 0.03 |
| Wing |  |  |  |  |  |  |  |  |  |
| WL* | 8.70 ± 0.17 | 8.63 ± 0.39 | 8.36 ± 0.28 | 8.66 ± 0.46 | 8.27 ± 0.89 | 6.95 ± 0.77 | 8.41 ± 0.21 | 8.59 ± 0.20 | 8.27 ± 0.37 |

*significant difference
