# Supplementary material for: Life history and chemical ecology of the Warrior wasp Synoeca septentrionalis (Hymenoptera: Vespidae, Epiponini)
Source: PLoS One. 2018 Mar 22;13(3):e0194689. doi: 10.1371/journal.pone.0194689 (PMC5864055; doi:10.1371/journal.pone.0194689)
Supplement: S1 Fig — (DOCX) [file pone.0194689.s002.docx]

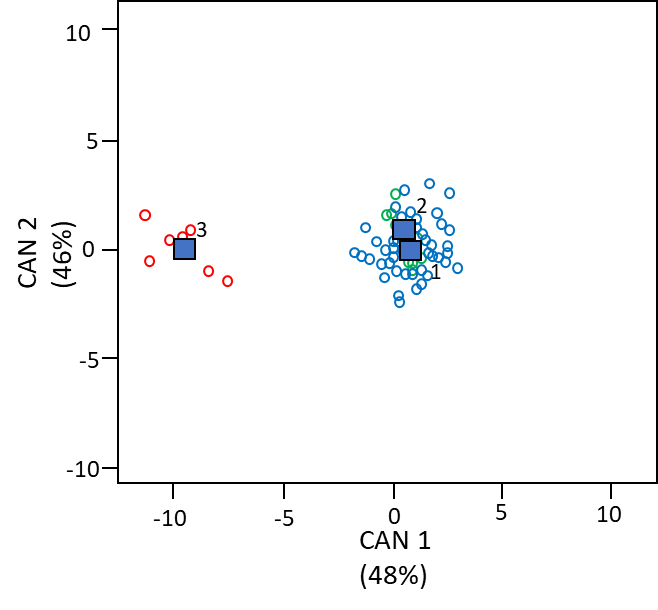


**S1 Fig.** **Discriminate analysis of *Synoeca septentronalis* adults.** Workers (1=blue), queens (2=green), and males (3=red) from MU1 colony. Showing the clear difference of the males from the queens and workers those results overlap.
